# Phenotypical Screening on Neuronal Plasticity in Hippocampal-Prefrontal Cortex Connectivity Reveals an Antipsychotic with a Novel Profile

**DOI:** 10.3390/cells11071181

**Published:** 2022-03-31

**Authors:** Michael Spedding, Claude Sebban, Thérèse M. Jay, Cyril Rocher, Brigitte Tesolin-Decros, Paul Chazot, Esther Schenker, Gabor Szénási, György I. Lévay, Katalin Megyeri, Jozsef Barkóczy, Laszlo G. Hársing, Ian Thomson, Mark O. Cunningham, Miles A. Whittington, Lori-An Etherington, Jeremy J. Lambert, Ferenc A. Antoni, Istvan Gacsályi

**Affiliations:** 1 Institut de Recherches Internationales Servier, 92284 Suresnes, France; estherschenker7@gmail.com; 2 Spedding Research Solutions SAS, 78110 Le Vésinet, France; 3 Hôpital Charles Foix, 94205 Ivry-sur-Seine, France; csebban@yahoo.fr (C.S.); brigittedecros@yahoo.fr (B.T.-D.); 4 INSERM UMR_S894, Hôpital Sainte-Anne, Université de Paris V Descartes, 75014 Paris, France; therese.jay@inserm.fr (T.M.J.); cyril.rocher@yahoo.fr (C.R.); 5 Department of Biosciences, University of Durham, Durham DH1 3LE, UK; paul.chazot@durham.ac.uk; 6 Behavioural Pharmacology Laboratory, EGIS Pharmaceuticals Ltd., 1106 Budapest, Hungary; szenasi.gabor@med.semmelweis-univ.hu (G.S.); gy.levay@richter.hu (G.I.L.); katalinmegyeri@yahoo.com (K.M.); barkoczy.jozsef@hungarnet.hu (J.B.); harsing.laszlo@med.semmelweis-univ.hu (L.G.H.J.); franzantoni@gmail.com (F.A.A.); istvan.gacsalyi@aurigon.eu (I.G.); 7 Institute of Translational Medicine, Semmelweis University, 1085 Budapest, Hungary; 8 Gedeon Richter Plc., 1103 Budapest, Hungary; 9 Department of Morphology and Physiology, Faculty of Health Sciences, Semmelweis University, 1088 Budapest, Hungary; 10 Hungarian Defence Forces Medical Centre, 1134 Budapest, Hungary; 11 Department of Pharmacology and Pharmacotherapy, Semmelweis University, 1085 Budapest, Hungary; 12 Institute of Neurosciences, Faculty of Medical Science, Newcastle University, Newcastle upon Tyne NE2 4HH, UK; contact@speddingresearchsolutions.fr (I.T.); mark.cunningham@tcd.ie (M.O.C.); 13 Discipline of Physiology, School of Medicine, Trinity College Dublin, D02 PN40 Dublin, Ireland; 14 Deceased, formerly of Hull York Medical School, University of York, Heslington HU6 7RX, UK; miles.whittington@hymns.ac.uk; 15 Ninewells Hospital and Medical School, University of Dundee, Dundee DD1 9SY, UK; l.etherington@dundee.ac.uk (L.-A.E.); j.j.lambert@dundee.ac.uk (J.J.L.); 16 Centre for Discovery Brain Sciences, Deanery of Biomedical Sciences, University of Edinburgh, Edinburgh EH8 9XD, UK; 17 ATRC Aurigon Toxicological Research Center Ltd., 2120 Dunakeszi, Hungary

**Keywords:** schizophrenia, EGIS 11150, S 36549, theta rhythm, phencyclidine, ketamine, dysconnectivity

## Abstract

Dysfunction in the hippocampus-prefrontal cortex (H-PFC) circuit is a critical determinant of schizophrenia. Screening of pyridazinone-risperidone hybrids on this circuit revealed EGIS 11150 (S 36549). EGIS 11150 induced theta rhythm in hippocampal slice preparations in the stratum lacunosum molecular area of CA1, which was resistant to atropine and prazosin. EGIS 11150 enhanced H-PFC coherence, and increased the 8–9 Hz theta band of the EEG power spectrum (from 0.002 mg/kg i.p, at >30× lower doses than clozapine, and >100× for olanzapine, risperidone, or haloperidol). EGIS 11150 fully blocked the effects of phencyclidine (PCP) or ketamine on EEG. Inhibition of long-term potentiation (LTP) in H-PFC was blocked by platform stress, but was fully restored by EGIS 11150 (0.01 mg/kg i.p.), whereas clozapine (0.3 mg/kg ip) only partially restored LTP. EGIS 11150 has a unique electrophysiological profile, so phenotypical screening on H-PFC connectivity can reveal novel antipsychotics.

## 1. Introduction

It is crucial to develop new approaches, related to the fundamental pathophysiological processes, in order to find new drugs in psychiatric disorders [1,2]. A major effort was put into the MATRICS (Measurement and Treatment Research to Improve Cognition in Schizophrenia), CNTRICS (Cognitive Neuroscience Treatment Research to Improve Cognition in Schizophrenia) and IMI-Newmeds initiatives to define new cognitive and imaging paradigms exploring the cognitive impairments in schizophrenia; the studies emphasise the necessity for new animal models if breakthroughs are to be found [3,4,5,6,7].

What hypotheses are available for novel therapies in schizophrenia? Schizophrenia has been claimed to be a disease of disconnection of key circuits. Wernicke in 1906 proposed disruption of association fibre tracts and Bleuler (1911) proposed splitting of different mental domains. Friston & Frith published in 1995 the disconnection hypothesis and that the neuroimaging of language in schizophrenics showed reduced frontotemporal coupling [8,9]. It is now accepted that mutations affecting neuronal plasticity, especially copy number variants (CNVs) may increase the odds ratio of developing schizophrenia [10] and support the conclusion that there are deficits in neuronal connectivity. There are major alterations in thalamocortical activation, with a marked reduction of the number of thalamic neurones projecting into the prefrontal cortex [11] and a reduced volume in the thalamus, which will lead to inappropriate activation of cortical areas and reduced blood flow in the prefrontal cortex. The hippocampal to ventromedial prefrontal cortex (H-PFC) pathway is now widely accepted to be a critical pathway in psychiatric disorders [7,12,13,14] and crucial for cognition; hippocampal memories are laid down in the ventromedial PFC with time in man [15], and H-PFC coupling is impaired in schizophrenia [16,17]. Indeed, H-PFC and thalamic miscommunication is the reason for working memory deficits in schizophrenia [18].

The H-PFC pathway is exquisitely sensitive to stress, where neuroplasticity and long-term potentiation (LTP) are blocked by exposure to a stressful environment [19]. Cognitive changes in schizophrenia may be due to stress-induced impairments in H-PFC circuits with increased activation of the amygdala [20]. Furthermore, Df(16)A^+/−^ mice, which model a human chromosome 22 (22q11.2) deletion syndrome, the strongest known genetic risk factor for schizophrenia with an odds ratio of 30, showed disruption in learning and H-PFC coherence, especially in the theta band [21]. It was argued that this may be a fundamental component of the pathophysiology underlying schizophrenia [22], encouraging precise drug targeting on this pathway, so we screened a series of 20 pyridazinone-risperidone hybrids specifically on H-PFC in an example of circuit-based phenotypic screening. EGIS 11150 (S 36549) stood out as being uniquely potent, but for unknown reasons.

There are multiple variables which may change the potency of a drug at a receptor, and the exact receptor profile for antipsychotic activity is unknown [23,24], so it is difficult to predict antipsychotic activity from a receptor profile, especially if much greater efficacy over standard drugs is the goal. EGIS 11150 was found to be highly active, and had an atypical antipsychotic receptor binding profile which had affinity, in decreasing order, for: α_1_-adrenoceptors > 5-HT_2A_ > 5-HT_7_ > α_2C_ > D_2_ receptors, with K_i_s of 0.5 nM, 3.1 nM, 9 nM, 13 nM and 120 nM, respectively [25]. A priori, such an approach has some risks in that the tests are not validated by previous drugs, but it is a way to have genuine innovation [1,26].

How can changes in cognition be correlated to disconnection, and how can these be remedied? Theta hippocampal EEG activity unites cell assemblies in the limbic system and is directly associated with memory processes [27,28]. Phase precession of theta rhythm coordinates the firing of place cells and entorhinal grid cells in various learning situations as well as exploratory activity in rats. Theta rhythm (4–8 Hz) is the rhythm which facilitates LTP, neuronal plasticity and learning [29] when increased in its upper band (6–8 Hz). Theta rhythm in the prefrontal cortex is increased by some but not all antipsychotics [30,31,32]. Increased theta rhythm in the frontal cortex of schizophrenic patients may indicate therapeutic responsiveness to clozapine [33,34], however scalp theta rhythm is a resultant of many sources and H-PFC contributes to only a minor extent. Buzsáki’s group has shown that H-PFC cell assemblies are linked by theta during maze learning with precession during the task [35] see also [36,37]. Thus if schizophrenia is a disconnection disease, cell assemblies linked by theta rhythm may be a direct measure of local connectivity, and would be particularly important in the stress-susceptible H-PFC pathway [21,22], thus screening for agents which increase theta rhythm in this pathway may allow the discovery of novel therapeutics.

The use of NMDA antagonists (phencyclidine, ketamine) in animals and man has been claimed to be an experimental surrogate of schizophrenia and NMDA antagonists reveal different pharmacology compared with challenges mediated by the dopaminergic stimuli of amphetamine or apomorphine [32,38,39,40]. Screening of functional NMDA antagonism was used in conjunction with theta rhythm (with coherence of the power spectrum between the hippocampal and the prefrontal cortex), PCP reversal, and stress-impaired LTP in H-PFC coupling. EGIS 11150 was found to have unique pharmacology, which could not be predicted using merely conventional screening for antipsychotic compounds. The present paper describes the electrophysiological profile of the drug, while the effects of the compound in radioligand binding assays, classical schizophrenia models and assays of cognitive performance have been previously published [25].

## 2. Methods and Materials

### 2.1. Drugs

All solutions were freshly prepared and mice were administered a volume of 10 mL/kg (ip. and sc.) or 20 mL/kg (p.o.). Rats received a volume of 5 mL/kg for p.o. and 2 mL/kg for ip. and sc. treatments.

EGIS 11150 (4-Chloro-5-{2-[4-(6-fluoro-benzo[d]isoxazol-3-yl)-piperidin-1-yl]-ethylamino}-2-methyl-2H-pyridazin-3-one;), risperidone and olanzapine were synthesized by EGIS Pharmaceuticals PLC. Budapest Hungary. Phencyclidine hydrochloride (PCP) was from Sigma (St. Louis MO, USA, or Tocris Biosciences, Bristol, UK), as were atropine and prazosin; ZD7288 and gabazine were from Tocris Biosciences (UK), and ketamine, haloperidol and clozapine from RBI (Natick, MA, USA). For the EEG experiments PCP, ketamine and the other drugs were dissolved in 0.9% NaCl containing agar. Phencyclidine hydrochloride was suspended in 0.4 % (*w*/*v*) hydroxypropyl methylcellulose solution (Methocell F4 M, Dow Chemical Company, Midland, MI, USA). The doses administered were calculated on the basis of the salt.

### 2.2. Animals

Experiments were performed in multiple laboratories, using protocols that had been extensively validated previously, for optimization of experimental conditions and of dosing regimes. Table 1 shows the comparative data on the animals and drugs used for each group, with the approval of the relevant ethical committees. Adult male Wistar rats (~150 g) and aged male Wistar rats (8 months) were used for the hippocampal slice preparations and the EEG recordings, respectively. Adult male Sprague Dawley rats (300–400 g) were used for platform stress and LTP recordings. All animals were maintained under temperature-controlled conditions (22 ± 2 °C), with a normal 12 h light dark cycle and ad libitum access to food and water.

Adult male C57/Bl6 mice (2–4 months) were obtained from Charles River UK, and used for platform stress and subsequent ex vivo LTP recordings (Table 1).

Male C57/Bl6N mice (Charles-River, Sulzfeld, Germany) were housed in humidity—(60 ± 10 %) and temperature-controlled (23 ± 1 °C) rooms with a 12 h light/dark cycle. Food and water were provided ad libitum. Behavioural testing was performed between 9 am and 2 pm.

Normally distributed data sets were compared using the paired Student’s *t*-test and unpaired Student’s *t*-test. Statistical significance between more than two normally distributed data sets was tested by one-way analysis of variance test (or two-way when specified), followed by a Newman-Keuls test to compare individual pairs of data. Non-Gaussian data sets were tested by non-parametric Mann-Whitney test. Indications of significance correspond to *p*-values < 0.05 (*), *p* < 0.01 (**) and *p* < 0.001 (***).

### 2.3. Hippocampal Slice Preparation: Oscillations

Adult male Wistar rats (~150 g) were anaesthetized with isoflurane, followed by an intramuscular injection of xylazine (~10 mg/kg) and ketamine (~100 mg/kg), before perfusion with oxygenated, sucrose-containing artificial cerebrospinal fluid (sACSF, Adelaide, SA, Australia). Horizontal slices of hippocampus (450 mm thick) were maintained at the interface between humidified carbogen gas (95% O_2_/5% CO_2_) and normal ACSF that contained (in mM): 126 NaCl, 3 KCl, 1.25 NaH_2_PO_4_, 1 MgSO_4_, 1.2 CaCl_2_, 24 NaHCO_3_ and 10 glucose at 34 °C. All drugs were bath-applied: EGIS 11150 (200 nM), ZD7288 (10 mM), gabazine (2 mM). Extracellular field potential recordings were taken with micropipettes (2–5 MΩ) filled with ACSF and band pass filtered at 0.002–0.5 kHz. A secondary band pass filter (0.003–0.013 kHz) was applied during post processing of data. Stable oscillations were recorded 1 h after EGIS 11150 application. Power spectra were derived from Fourier analysis of 60-s epochs of data.

### 2.4. EEG, In Vivo

#### 2.4.1. EEG Electrode Implantation

Electrodes were implanted under chloral hydrate (350 mg/kg i.p.) anesthesia, using a stereotaxic frame. Using the Bregma suture as reference, two transcortical bipolar electrodes were implanted into the right and the left prefrontal cortex (PFC) at A +4 mm and L 2.5 mm [30,31]. Two transcortical electrodes were implanted into the right and the left sensorimotor cortex (SMC) at A −4 mm and L 2.5 mm and two electrodes (3 mm) were implanted into the hippocampal cortex (HIC) at A −2 mm and L 2.5 mm [41]. Animals were earthed via a stainless steel screw fixed onto the frontal bone. After the connection of electrodes and the screw to a connection plug, they were fixed to the skull using an acrylic cement. Recordings were carried out by placing the rat in a Perspex cylinder which did not allow the rat to turn, but did not tightly constrain the animal The cylinder was placed into a large electrically and acoustically insulated chamber. A light source (60 W) was applied at a distance of 10 cm in front of the rat’s nose to keep the animal with widely opened eyes and the head held up (for recording details see Supplementary Materials). After 10 days of recovery, animals were gradually habituated to the restraining cage used during the 3 h EEG recordings to decrease artefacts of movement [30,31]. In general, no attempts of escape or notable stress reaction were observed.

#### 2.4.2. Data and Statistical Analysis

EEG Group size was 6 animals for each experiment. Animals for EEG recordings have been bred in-house from an outbreed colony of rats [30,31]. These animals were housed singly. Animals were injected with saline solution 24 h before receiving the drug injection, and the EEG power ratio calculated for each animal by subtracting the signal from the saline-treament from the drug-treatment, so only the effect of the drug was shown. The animals were randomized for drug treatments.

The ratios describing drug or challenge (single dose) effects over each 5 min period have been submitted to a one-way analysis of variance (ANOVA). A two-way ANOVA was used for dose-response analyses. The confidence interval corresponds to the error bars in each figure (*p* < 0.05). The power variations of each component (1–30 Hz) is considered significant when above or below 1. Vertical bars for each Hz show 95% confidence intervals.

### 2.5. Inhibition of H-PFC LTP Caused by Platform Stress

The procedure is fully described previously [19]. The stress protocol consisted in placing the rats on an elevated and unsteady platform for 30 min. The animal showed behavioural “freezing”, defecation and sometimes urination. At the end of stress, rats were anaesthetized (60 mg/kg i.p. pentobarbital) on the platform and immediately after, placed in the stereotaxic frame. High frequency stimulation (HFS) was administered to induce LTP, after preparation and stabilisation of the animals, 180 min after stress; drugs (EGIS 11150, clozapine or haloperidol) were administered. 40 min before HFS. PSP amplitudes were analysed using A/Dvance software, expressed as a percentage change of the mean response over a 30 min baseline period and presented in figures as the mean ± SEM for 2 min epochs. Electrophysiological data were averaged in consecutive 30 min periods (T0–30 min, T30–60 min, T60–90 min, T90–120 min) after LTP induction. Recording electrodes were positioned in the prelimbic cortex and a bipolar concentric stainless steel stimulating electrode was lowered into the ipsilateral CA1/subicular region of the ventral hippocampus. Test pulses (100 µs) were delivered every 30 s at an intensity that evoked a response of 70% of its maximum. High frequency stimulation (HFS) consisted of two series of 10 trains (250 Hz, 200 ms) at 0.1 Hz, 6 min apart, delivered at test intensity. Statistical comparisons were carried out using analysis of variance according to Rocher et al. (2004) [19]. In all cases, differences between groups were considered to be significant if *p* < 0.05.

### 2.6. Mouse Hippocampal Slice Preparation, Influence of Stress on LTP

Hippocampal slices (400 μm) were obtained from 2–4 month-old male C57/BL6 mice and incubated for at least 60 min. in artificial cerebrospinal fluid (aCSF) of the following composition: (in mM): NaCl, 124; KCl, 3; CaCl_2_, 2; NaHCO_3_, 26; NaH_2_PO_4_, 1.25; D-glucose, 10; MgSO_4_, 1; pH 7.4 with 95% O_2_/5% CO_2_.

Ex vivo extracellular recording from hippocampal CA1 pyramidal neurons. A hippocampal slice was placed in a submerged recording chamber and continuously perfused with aCSF at 31 °C. To induce and monitor basal synaptic transmission a bipolar stimulating electrode was positioned in stratum radiatum, allowing orthodromic stimulation of the Schaffer collateral-commissural pathway. Recordings were made in the CA1 dendritic layer using glass microelectrodes.

Stimulation was delivered at 0.033 Hz and the stimulus adjusted to produce a response 40% of the maximum fEPSP slope. For LTP experiments, maximal, or sub-maximal LTP was induced by a TBS protocol (4, or 3 pulses respectively delivered at 100 Hz, repeated 10 times, at an interval of 200 ms). The stimulus parameters, the acquisition and the analysis of fEPSPs were under the control of LTP software (courtesy of Dr. Anderson and Prof. Collingridge, Bristol University, UK; http://www.ltp-program.com, accessed on 27 February 2022). Statistical analysis of LTP was performed using SPSS statistical software. Comparisons of the PTP fEPSP values were measured using unpaired *t*-tests and the magnitude of LTP was measured by means of repeated measures linear regression analysis at 50–60 min after the TBS [42].

Stable baseline recordings of fEPSPs (0.033 Hz, monitored as the slope of the fEPSP on-line) were obtained for a minimum of 10 min. prior to experimentation. E11150 was perfused onto the brain slice for 20 min. prior to TBS and the slope of the fEPSP observed throughout the experimental period. The drug was then continually perfused for a further 60 min. throughout the remainder of the experiment.

Acute stress was elicited by placing the mice on an open elevated platform for 45 min. immediately prior to sacrifice as previously described [42].

### 2.7. Reversal Learning in the Rewarded T-Maze after Sub-Chronic Treatment with Phencyclidine (PCP)

Male C57Bl/6J (20–25 g b.w.) mice were housed (five animals/cage) and received a single injection of 3 mg/kg PCP or saline i.p. for two five-day periods separated by a break of two days. Three days after the last injection of PCP, the protocol of Deacon and Rawlins [43] was followed to habituate the animals to the test apparatus and to adapt them to supplementing their food intake with condensed milk from feeder cups placed at the end of the arms of the T-maze. Subsequently, the animals were allowed six learning trials consisting of 10 runs each in the T–maze per day with 5 sec intervals [44]. Only the right arm of the maze was baited during learning trials. After the sixth learning trial, eight animals (two from each treatment group) were tested for reversal learning on a single day in four trials of 10 runs 5 sec apart, inter-trial interval 40 min, during which only the left arm of the maze was baited [45]. The number of correct choices was recorded and statistically analysed. Vehicle or Egis-11150 (0.01 or 0.03 mg/kg) was administered p.o. 1 h before the commencement of reversal trials. The first group of eight animals was tested 24 h after the last learning trial and the other groups were examined over the next four days. As the treatment trends were similar on each day of testing the data from the five reversal trials are reported together.

## 3. Results

### 3.1. Effects on Theta Rhythm in Hippocampal Slices

**Figure 1:**
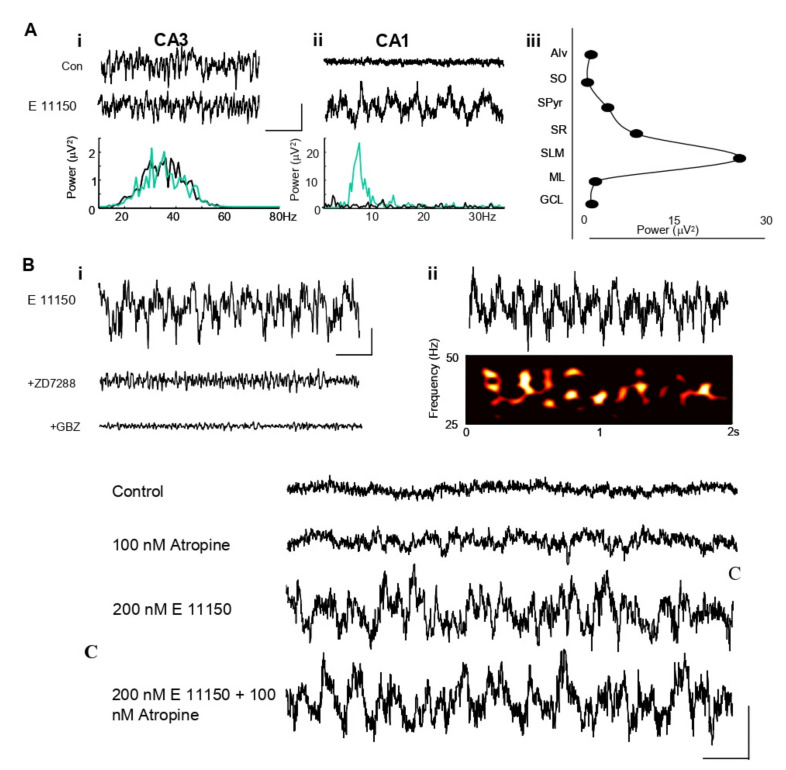
EGIS 11150 selectively generates a theta rhythm de novo in CA1 of hippocampal slices in vitro. (**A**). (**i**) Extracellular recordings from CA3 stratum radiatum show no effect of EGIS 11150. (**ii**) In stratum lacunosum molecular area of CA1, EGIS 11150 produces a persistent theta frequency rhythm. Scale bars 50 µV 200 ms. (**iii**) Laminar profile of CA1 theta, recorded in the layers indicated with extracellular electrode shows a highly localised occurrence in stratum lacunosum. Aalveus (Alv), Stratum oriens (SO), Stratum pyramidale (SPyr), Stratum radiatum (SR), Molecular layer dentate gyrus (ML), Granule cell layer dentate gyrus (GCL). (**B**). (**i**) EGIS 11150-generated theta rhythms were blocked by GBZ (2 µM) and ZD7288 (10 µM). (**ii**) Spectogram of the EGIS 11150-generated gamma rhythms. (**C**) EGIS 11150 induced theta rhythm is not affected by a muscarinic component in hippocampal region CA1 in vitro. Extracellular recordings from CA1 stratum lacunosum show 200 nM EGIS 11150 generates a persistent theta frequency rhythm, which is unaffected by the muscarinic agent, atropine, at a concentration of 100 nM. Scale bars 50 µV 200 ms.

EGIS 11150 (200 nM) generated a robust, persistent oscillatory rhythm in the CA1 area of the hippocampus in vitro (Figure 1A), representative of 6 experiments. Maximal mean power in the field was 25 ± 4 µV^2^ (*n* = 6) and frequency 6.4 ± 0.7 Hz. The oscillatory rhythm was very spatially localised, being seen only in stratum lacunosum and distal stratum oriens of area CA1 (Figure 1A(iii)). By contrast, spontaneous gamma frequency field potential rhythms in area CA3 were not significantly affected by this concentration of EGIS 11150. Mean powers in the band 25–50 Hz were: control 2.3 ± 0.5 µV^2^, EGIS 11150 2.6 ± 0.9 µV^2^ (*n* = 6, *p* > 0.1). The I_h_ blocker ZD7288 abolished the field oscillatory rhythm (Figure 1B(i)). Similarly, abolition of GABA_A_ receptor-mediated local synaptic inhibition with gabazine also abolished the rhythm, but blockade of muscarinic cholinoceptors with atropine (100 nM) had no effect (Figure 1C), as did blockade of α_1_-adrenoceptors with prazosin (data not shown). The EGIS 11150-generated rhythm was associated with theta-frequency amplitude modulation of on-going gamma rhythms (Figure 1B(ii)). Preliminary data suggest that the ability of EGIS 11150 to generate theta in the hippocampus de novo is shared by clozapine, but only when at least two orders of magnitude higher concentrations were used. Theta generation was not seen at any concentration of risperidone up to 2 µM.

The effects of EGIS 11150 were resistant to atropine in the hippocampal region CA1 in vitro. In the presence of 200 nM EGIS 11150 alone, the maximal mean amplitude in the field was 8.81 ± 2.30 mV^2^/Hz^2^ and frequency was 4.91 ± 0.18 mV^2^/Hz^2^. The addition of 100 nM atropine did not significantly affect the peak amplitude, 5.29 ± 1.29 mV^2^/Hz^2^, or the frequency, 5.44 ± 0.18 mV^2^/Hz^2^ of the EGIS 11150 induced theta rhythm (*n* = 8, *p* > 0.1).

### 3.2. Effects of EGIS 11150 on EEG at 8–9 Hz

**Figure 2:**
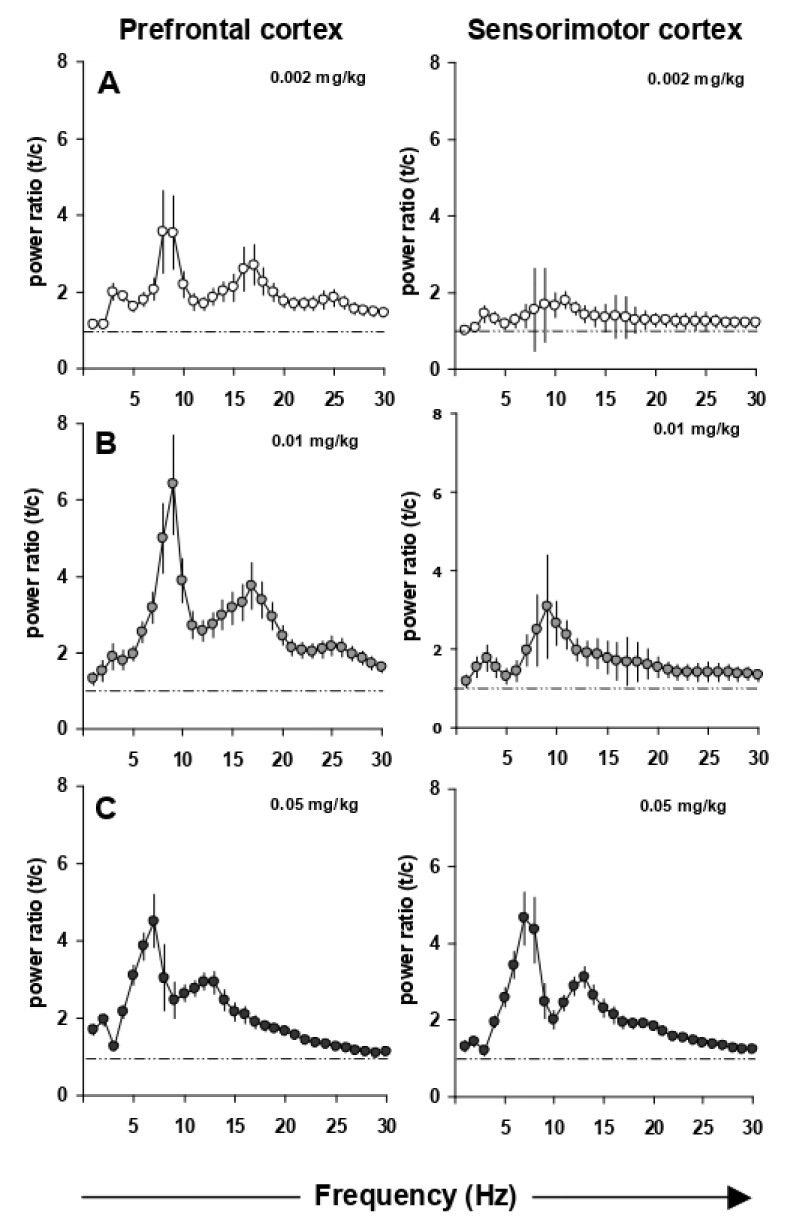
Power spectra (total power) in the prefrontal and sensorimotor cortex with increasing doses of EGIS 11150. Panel (**A**) 0.002 mg/kg EGIS 11150 induces high theta bands (8–10 Hz) in the prefrontal cortex. Panel (**B**) 0.01 mg/kg EGIS 11150 maximally induces high theta bands in the prefrontal cortex. Panel (**C**) 0.05 mg/kg EGIS 11150 maximally induce high theta bands in the sensorimotor cortex (*n* = 6).

**Figure 3:**
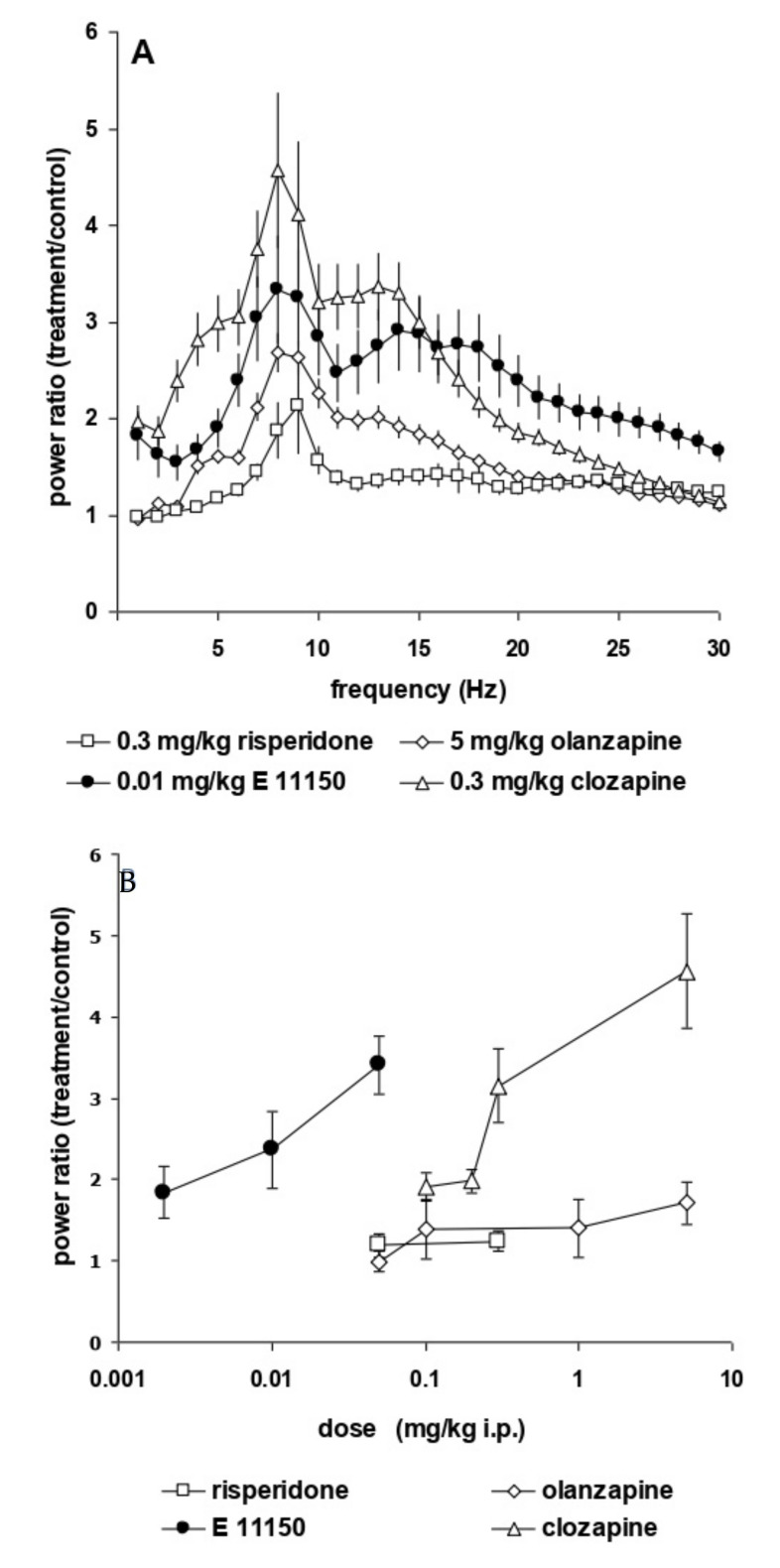
Comparison of the effects of EGIS-11150 on the EEG power spectrum with those of risperidone, olanzapine and clozapine. Panel (**A**) Composite graph of power spectra (total power) of the prefrontal cortex after administration of 0.3 mg/kg i.p. risperidone 5 mg/kg i.p. olanzapine, 0.3 mg/kg s.c. clozapine and 0.01 mg/kg i.p. EGIS-11150. Panel (**B**) Dose-response relationship of the effects of antipsychotics on total EEG power in the prefrontal cortex (*n* = 6).

EEG power spectra remained stable over time. Maximally 15% changes in power over the entire frequency range were observed with saline administrations performed two days apart. Figure 2 shows the dose-dependent increase in the upper theta rhythm of the prefrontal and sensorimotor cortex power spectra. This induction is observed already at the lowest i.p. dose of 0.002 mg/kg EGIS 11150 and reaches a maximal effect at 0.01 mg/kg (Figure 2B). The increase of theta rhythm by EGIS 11150 is 30 to 100-fold more potent than that observed with clozapine, olanzapine or risperidone (Figure 3A,B). Risperidone has the least effect on the power spectrum, whereas clozapine changes of the spectrum closely resemble those observed with EGIS 11150.

### 3.3. EGIS 11150 Rescues PCP—and Ketamine-Induced Effects on EEG

**Figure 4:**
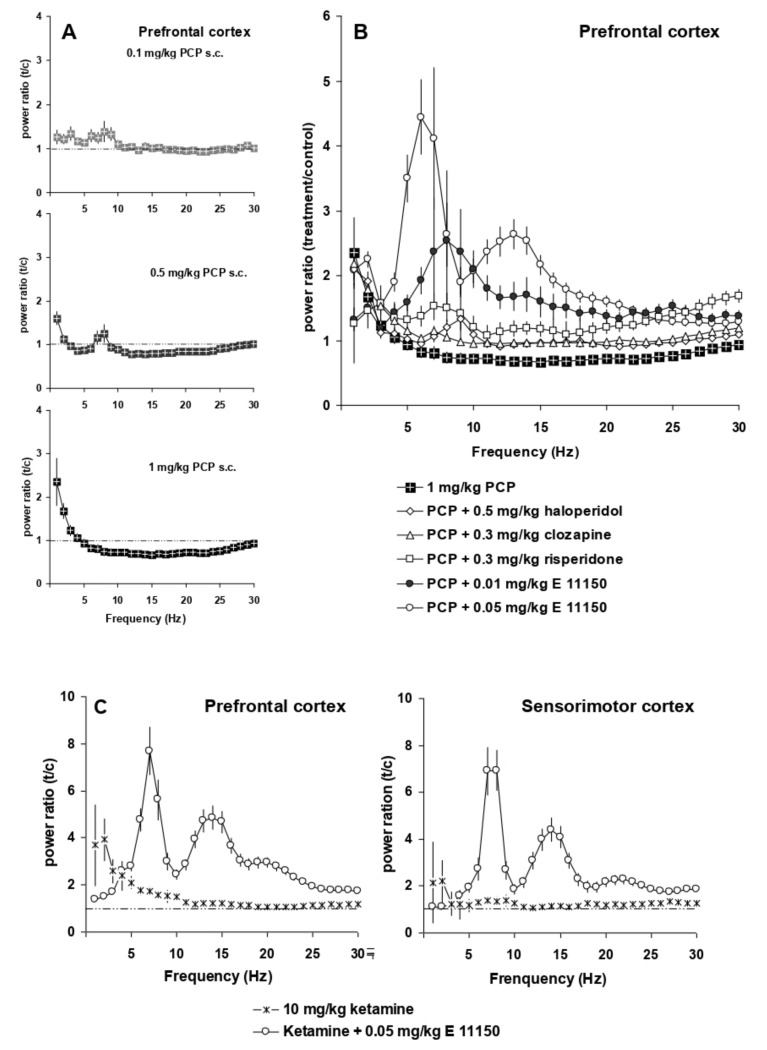
Interaction of antipsychotic compounds with NMDA antagonists in the prefrontal cortex. Panel (**A**) EEG power spectra induced by increasing doses of PCP. Panel (**B**) Composite graph of power spectra (total power) of 1 mg/kg s.c. PCP, as well as 0.3 mg/kg s.c. clozapine, 0.5 mg/kg i.p. haloperidol, 0.3 mg/kg i.p. risperidone, 0.01 mg/kg i.p. EGIS 11150, 0.05 mg/kg i.p. EGIS-11150 in the presence of PCP. Panel (**C**) Power spectra (total power) of the prefrontal and sensorimotor cortex of 10 mg/kg i.p. ketamine and 0.05 mg/kg i.p. EGIS-11150 in the presence of ketamine (*n* = 6).

The NMDA receptor antagonist, phencyclidine, induced at very low doses (0.1–3 mg/kg s.c) a unique power spectrum with strong changes in total power as previously reported [32]. The two lowest doses, 0.1 and 0.5 mg/kg s.c., caused small increases in EEG power at low frequencies (1–3 and 8 Hz; Figure 4A). At higher frequencies (9 to 30 Hz), the power spectrum shows desynchronization, which is more marked (30% ± 4% reduction in power) at 1 mg/kg. At 1 mg/kg s.c., there is a strong increase in EEG power (2.4 ± 0.4 fold) at 1 Hz.

The atypical antipsychotics clozapine and risperidone, as well as the classical haloperidol prevented the desynchronization effect of PCP (1 mg/kg s.c.) on the power spectra of the prefrontal cortex in conscious, restrained rats (Figure 4B). Significantly, the effects of EGIS 11150, at the dose of 0.05 mg/kg on theta rhythm (7–9 Hz) were similar to control conditions (Figure 3) being unimpaired by PCP (1 mg/kg s.c.); the effects of 0.01 mg/kg were also resistant to PCP (Figure 4B).

Ketamine produces positive symptoms associated with schizophrenia, as well as disturbances in working memory and attention on man [38]. When give at 10 mg/kg i.p., it synchronised the EEG power spectrum in the conscious rat at low frequencies ranging from 1–5 Hz, but there was no evidence of desynchronisation. In the presence of ketamine, EGIS 11150, at the dose of 0.05 mg/kg i.p., showed its characteristic profile, i.e., it fully opposed the effects of ketamine in the conscious restrained rat (Figure 4C).

### 3.4. EEG Coherence

**Figure 5:**
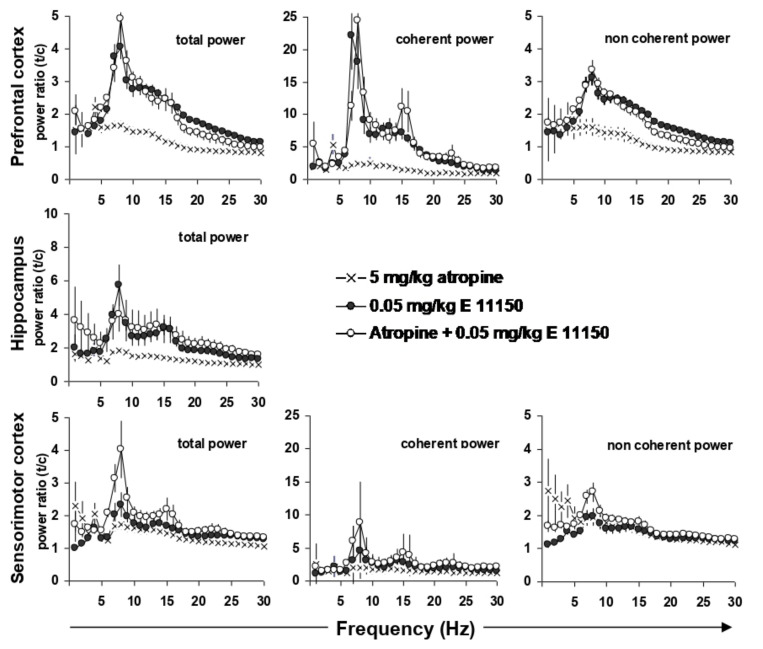
Effects of EGIS-11150 on EEG power in prefrontal cortex, in hippocampus and on hippocampal to prefrontal cortex coherence. Note the marked increase in hippocampal-prefrontal cortex coherence. Atropine (5 mg/kg i.p.) did not modify the effects of EGIS 11150 (0.05 mg/kg) indicating that the effects of EGIS 11150 on theta were presumably not of cholinergic origin; note however, that there was a small increase at 4 Hz in the presence of EGIS 11150 and atropine. Effects on sensorimotor cortex were considerably less marked. The 95% confidence interval corresponds to the error bars in each figure, *n* = 6.

When the EEG power effects of EGIS 11150 (0.05 mg/kg i.p.) in the PFC are expressed as coherent, i.e., hippocampal activity dependent, and non-coherent power, the increase in the upper theta rhythm appeared to be mainly related to hippocampal activity (Figure 5). Indeed, besides inducing a 5-fold increase in total power, coherent power was increased by EGIS 11150 (0.05 mg/kg i.p.) more than 20-fold. The non-coherent, hippocampus-independent, power showed an increase of only 3–5-fold. This strongly suggests that EGIS 11150 reinforces the hippocampal to PFC relationship for the upper theta rhythm.

The theta rhythm in the EEG of conscious rats is partially modified by atropine. Atropine, a nonselective muscarinic antagonist, induces a small increase in the lower theta band, whereas procognitive agents induce the higher theta bands. Atropine, administered at a dose of 5 mg/kg i.p., increased theta power in the prefrontal and sensorimotor cortex at 4 Hz (Figure 5). When expressed as coherent power, which relates network properties between the hippocampus and the prefrontal or sensorimotor cortex, the 8 Hz power was increased by 5-fold in respect to the total prefrontal power. Simultaneous injection of atropine and EGIS 11150 (0.05 mg/kg i.p.) shifted the increase of theta power from 4 to 8 Hz in the prefrontal and sensorimotor cortex, as well as the hippocampus.

These results suggest that EGIS 11150 might also have a cholinergic component, besides modulating glutamatergic effects, as demonstrated by the rescue of PCP—and ketamine-induced changes.

### 3.5. Effects of EGIS 11150 on the Inhibition of H-PFC LTP Caused by Platform Stress In Vivo

**Figure 6:**
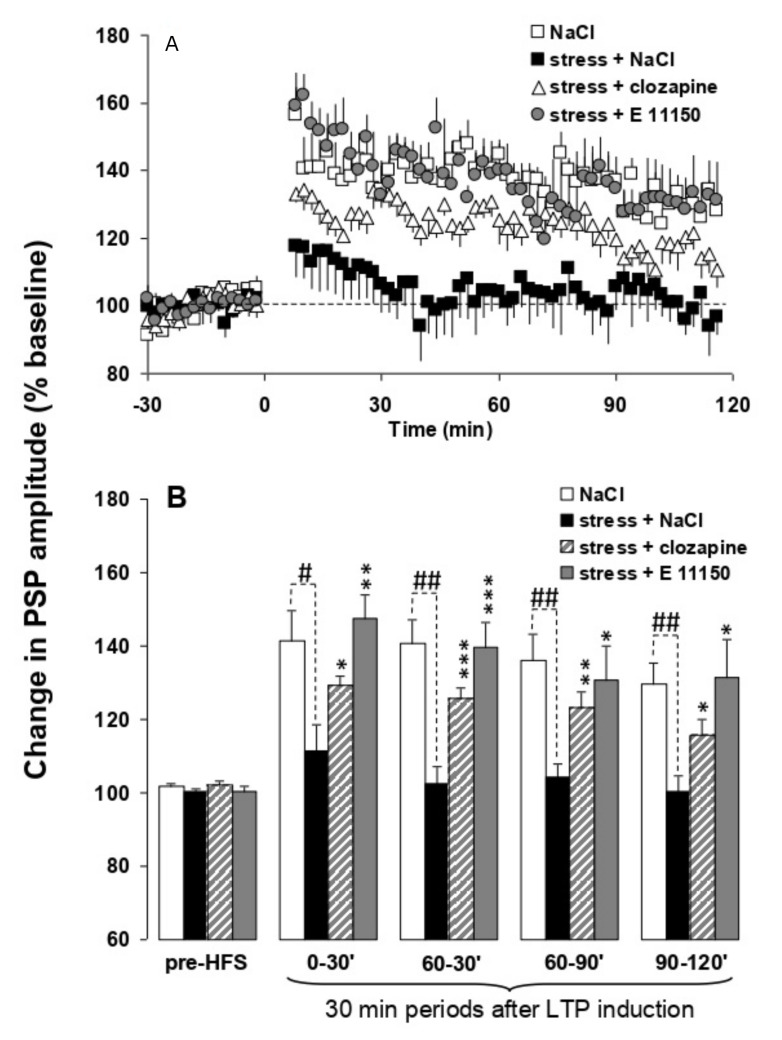
Comparison of the effects of EGIS 11150 and clozapine on stress-induced impairment of hippocampal-prefrontal cortex LTP in vivo. Panel (**A**) EGIS 11150 administered at 0.01 mg/kg and clozapine at 0.3 mg/kg after stress, 40 min prior to HFS enables the induction of LTP in stressed rats when compared to saline-pretreated stressed rats. Values are mean ± SEM of the normalized postsynaptic potential (PSP) amplitude. Panel (**B**) LTP in saline, EGIS 11150 and clozapine pretreated stressed rats represented at different time periods. PSP amplitudes were analysed using A/Dvance software, expressed as a percentage change of the mean response over a 30 min baseline period and presented in figures as the mean ± SEM for 2 min epochs. Electrophysiological data were averaged in consecutive 30 min periods (T0–30 min, T30–60 min, T60–90 min, T90–120 min) after LTP induction. Statistical comparisons were carried out using analysis of variance [19]. The first and following groups of columns represent 30 min periods of mean ± SEM of the normalized PSP amplitude before, and after HFS. ANOVA: * *p* < 0.05 ** *p* < 0.01 and *** *p* < 0.001 vs. saline-treated stressed rats, ANOVA: ^#^ *p* < 0.05, ^##^ *p* < 0.01, vs. saline-treated stressed rats.

**Figure 7:**
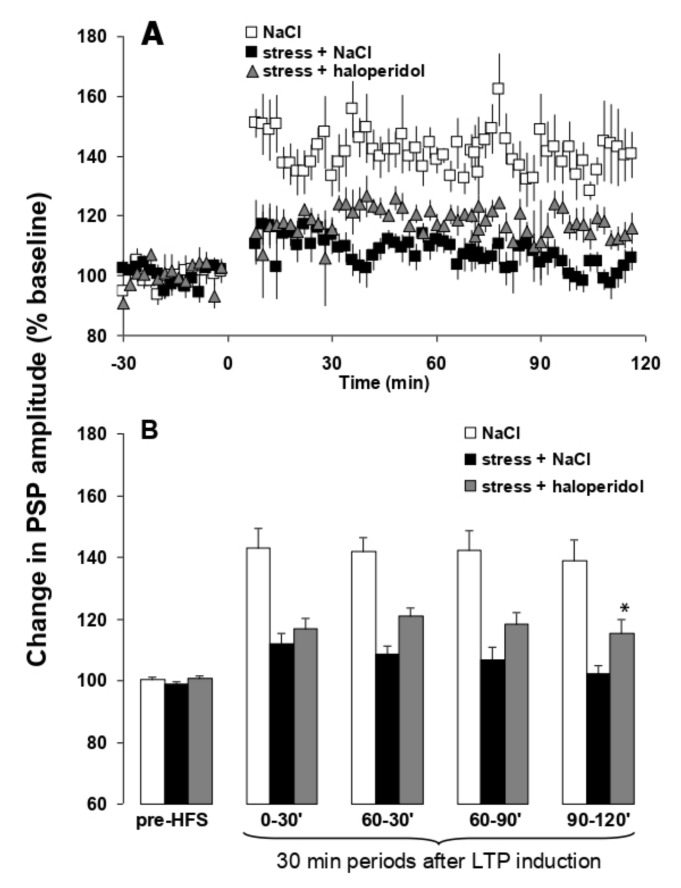
Haloperidol does not fully prevent stress-induced impairment of hippocampal-prefrontal cortex LTP in vivo. Panel (**A**) LTP in stressed rats treated with haloperidol is not significantly different from saline-treated stressed rats. Values are mean ± SEM of the normalized postsynaptic potential (PSP) amplitude. HFS is indicated by arrows. Panel (**B**) LTP in saline and haloperidol treated stressed rats represented at different time periods. The first and following groups of columns represent respectively 30 min periods of mean ± SEM of the normalized PSP amplitude before, and after HFS. ANOVA: * *p* < 0.05 vs. saline-treated stressed rats.

Theta burst stimulation of the hippocampal subiculum pathway to medial prefrontal cortex (H-PFC) induces long lasting stable LTP in anaesthetised rats, which have not been stressed. Thirty minute exposure to platform stress abolishes H-PFC LTP, for at least 4 h, if the rats are anaesthetised after the elevated platform stress (Figure 6) [19]. The dose of 0.01 mg/kg i.p. EGIS 11150 administered after the stress, and the rats were anaesthetized, fully reversed the inhibition of H-PFC LTP induced by platform stress. Clozapine was less efficacious, and only partially restored LTP at the same dose which induced robust theta rhythm (0.3 mg/kg i.p.; Figure 6), and haloperidol (1 mg/kg, i.p., Figure 7) was scarcely active.

### 3.6. Effects of EGIS 11150 on Submaximal LTP Recorded from In Vitro Hippocampal Slices of Adult Mice

**Figure 8:**
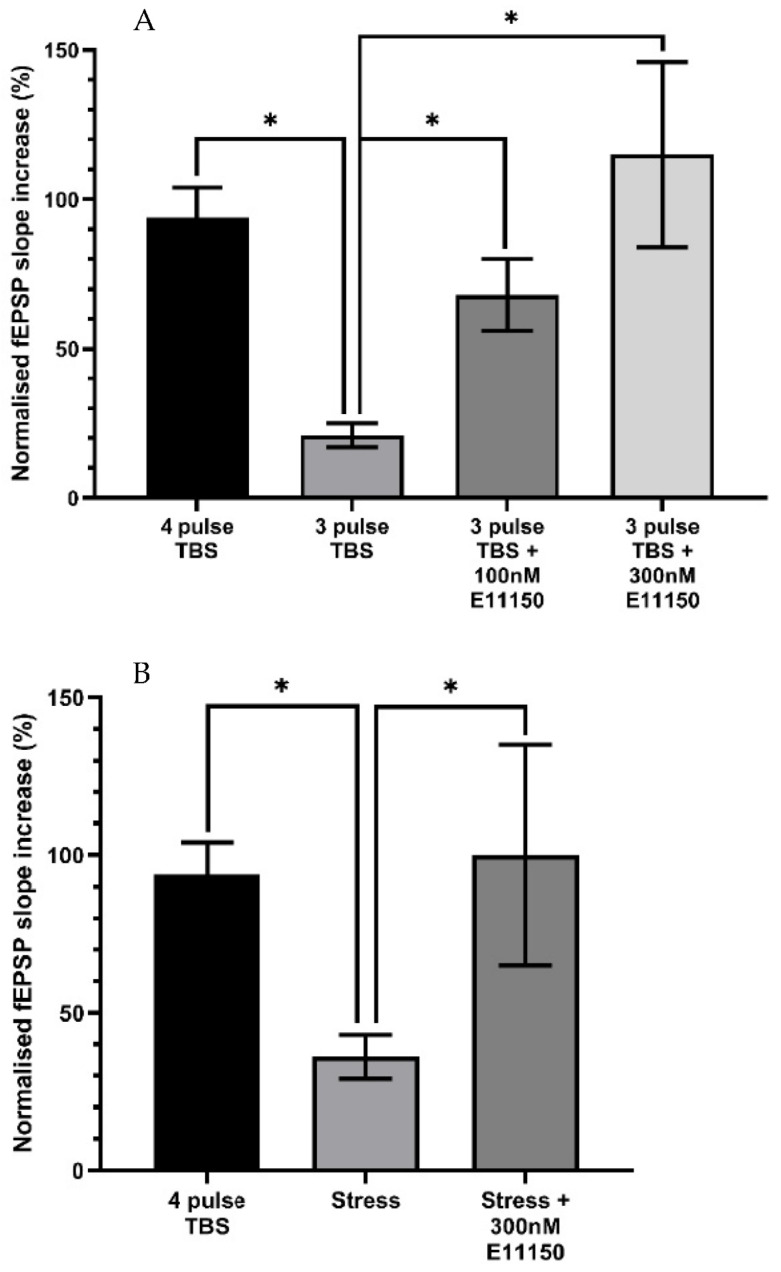
Application of E11150 to mouse hippocampal slices enhances submaximal and stress impaired LTP recorded from CA1 pyramidal neurones. Panel (**A**) The histogram illustrates that the magnitude of LTP (expressed as the percentage increase of the fEPSP slope) induced by the delivery of a 3-pulse TBS is significantly less than that resulting from delivery of a 4-pulse TBS protocol. The bath application of E11150 (100 nM and 300 nM) significantly enhanced the magnitude of LTP induced by the 3-pulse TBS. Each column represents the mean ± SEM of 4–11 individual experiments (* *p* < 0.05). Panel (**B**) A prior episode of acute stress to mice decreased the magnitude of ex vivo CA1 pyramidal neuron LTP induced by a 4-pulse TBS, in comparison to that induced by a 4-pulse TBS, recorded from equivalent CA1 neurones obtained from control mice. The bath application of E11150 (300 nM) significantly enhanced the magnitude of stress impaired LTP. Each column represents the mean ± SEM of 4–12 individual experiments * *p* < 0.05).

Delivery of a 4-pulse theta-burst stimulation (TBS) protocol to the CA1 dendritic region of hippocampal slices induced an initial post-tetanic potentiation (PTP) followed by a robust form of LTP determined 50 min post-TBS. A 3-pulse TBS protocol resulted in a significantly reduced magnitude of LTP at 50 min post-TBS (4 pulse control = 94 ± 10% increase; *n* = 12; 3 pulse control 21 ± 4% increase; *n* = 11, *p* < 0.05) Figure 8A. The effect of E11150 (100–300 nM) on sub-maximal LTP induced by a 3-pulse TBS was determined following a 20 min pre-incubation period with the drug (Figure 8). Application of either 100 nM or 300 nM E11150 resulted in a significant enhancement of the magnitude of LTP assessed 50 min after delivery of the 3 pulse TBS (3 pulse control = 21 ± 4% increase; *n* = 11; 100 nM E11150 = 68 ± 12% increase; *n* = 7; 300 nM E11150 = 115 ± 31%; *n* = 4, *p* < 0.05). E11150 (100 nM–300 nM) had no effect on basal transmission (*p* > 0.05). The data indicates that E11150 facilitates LTP in the CA1 region of the hippocampus, probably by reducing the threshold for its induction.

### 3.7. Acute Stress Suppresses Ex Vitro Hippocampal CA1 LTP In Vitro, a Deficit Reversed by EGIS 11150

We examined whether EGIS 11150 can modulate in vitro the synaptic plasticity of a mouse subjected to acute stress (45 min prior to sacrifice). As reported previously [42], this protocol produced a significant reduction in the magnitude of ex vivo LTP induced by the 4-pulse TBS protocol (4 pulse TBS control = 94 ± 10% increase; *n* = 12; “stressed” = 36 ± 7% increase; *n* = 12, *p* < 0.05)—Figure 8B. The recordings were made up to six hours after the platform paradigm and subsequent preparation of the hippocampal slice, indicating that the attenuation of LTP is long-lasting and well-maintained ex vitro (Figure 8B). Application of 300 nM EGIS 11150 resulted in a complete recovery of LTP when applied to hippocampal slices derived from previously stressed mice (“stressed” = 36 ± 7% increase; *n* = 12; “stressed” + 300 nM EGIS 11150 = 100 ± 35%; *n* = 4, *p* < 0.05)—Figure 8B.

### 3.8. Reversal Learning in the Rewarded T-Maze

**Figure 9:**
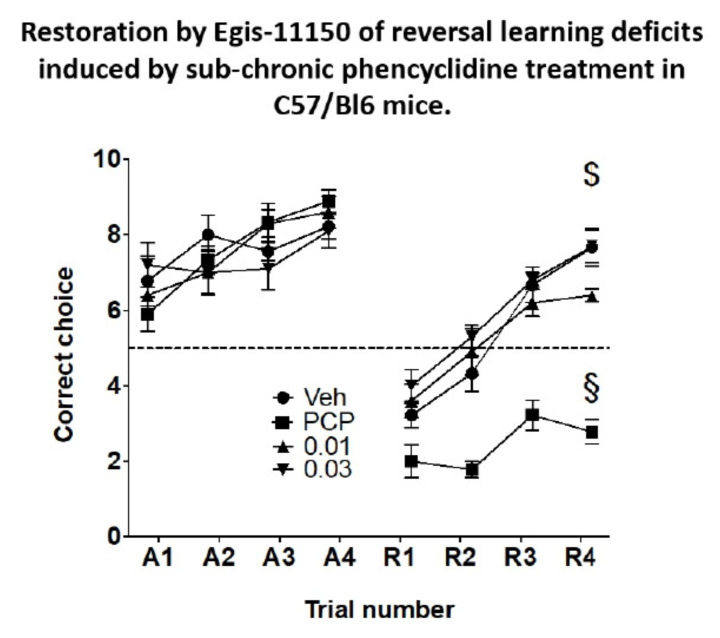
Impaired reversal learning by C57/Bl6 mice subjected to sub-chronic treatment with PCP is ameliorated by acute administration of Egis-11150 (0.03 and 0.1 mg/kg p.o.). Error scores of four acquisition sessions (left panel) and four reversal sessions (right panel) are shown. Data are mean ± SEM *n* = 10/group. Two-way ANOVA gave significant interaction drug treatment and trial number: F (9, 136) = 2.998, *p* = 0.0027. Dunnett’s post-hoc test: ^$^ *p* < 0.001 for both 0.03 and 0.1 doses vs. the respective PCP treated group acutely receiving vehicle, ^§^ *p* < 0.001 when compared to the group receiving sub-chronic as well as acute treatment with vehicle. (A = acquisition trials, R = reversal trials).

Over the course of 6 learning trials the PCP-treated animals eventually achieved the same mean correct choices in the test trials as the vehicle-treated controls (Figure 9). During the course of reversal learning the performance of animals subchronically as well as acutely treated with vehicle improved markedly (Figure 9). In contrast, the performance of the animals treated with PCP subchronically and acutely injected with vehicle was poor and significantly different from the vehicle-treated controls (Figure 9). Importantly, both doses of acutely administered Egis-11150 (0.03 and 0.1 mg/kg p.o.) countered the effect of PCP treatment and improved performance to levels indistinguishable from that of the vehicle treated group. The same doses of Egis-11150 had no discernible effect on reversal learning in intact mice (Figure S1).

## 4. Discussion

EGIS 11150 has an unusual receptor profile showing receptor affinities in decreasing order: α_1_-adrenoceptors > 5-HT_2A_ receptors > 5-HT_7_ receptors > α_2C_ receptors > D_2_ receptors (K_i_: 0.5 nM; 3.1 nM; 9 nM; 13 nM; 120 nM; [25,46]. The role of antagonism of α_1_-adrenoceptors, 5-HT receptors or D_2_-receptors by antipsychotic drugs has been debated but no absolute consensus has been arrived at for the profile of the ideal agent. However, there are so many confounding factors in drug-receptor interactions [23] that it is difficult to predict which receptor profile is responsible for the therapeutic efficacy of the existing antipsychotic agents. EGIS 11150, for unknown reasons, stood out in the various paradigms chosen to reflect the potential therapeutic benefit in schizophrenia. EGIS 11150 at 10µg/kg, i.p. has (i) shown benefits in cognitive paradigms [25,46], (ii) induced an increase in the theta rhythm in the hippocampus and medial prefrontal cortex associated with a strong reinforcement of the H-PFC relationship, (iii) reversed stress-induced inhibition of plasticity, as assessed by LTP in the H-PFC axis, and (iv) reversed PCP- and ketamine-induced cortical power desynchronization.

The finding that the compound also directly increased CA1 oscillatory rhythm in hippocampal slices, where both α_1_-adrenoceptor agonists and antagonists do not (Whittington, personal communication), confirms the originality of this finding. A CA1-specific local theta generator has been characterised previously with origin in stratum oriens [47]. Despite the different laminar origin, the oscillatory rhythm generated by EGIS 11150 appeared to have a similar underlying mechanism and may thus contribute to the development of theta rhythm. Furthermore, as with theta rhythms observed in vivo in hippocampus [48], this rhythm could entrain gamma rhythm in slices. Prazosin, a α_1_-adrenoceptor antagonist, induces a different profile in EEG studies using PCP-disruption, than EGIS 11150 [32]. It is unclear if the effects of EGIS 11150 are due to a unique combination of the known receptor affinities or to some other non-characterised effect of the agent. The unpublished structure-activity relationships indicate that α_1_-adrenoceptor antagonism is necessary, but by itself insufficient. The compound shows typical antipsychotic effects at higher doses [46], and at higher doses still (3 mg/kg p.o.) extrapyramidal side effects, presumably because of the weak D_2_ affinity.

Oscillations in the theta band have been found to increase with cognitive demands and the increase in temporofrontal theta activity during successful encoding is consistent with physiological evidence concerning the role of theta in gating LTP [35,49,50].

H-PFC connectivity occurs by a specific unidirectional glutamatergic tract in rodents [51] locking onto interneurons in the prefrontal cortex to give specific control [52]. In a review of this pathway, Colgin [53] showed that ~40% of PFC neurons are phase locked to CA1 theta rhythms, increasing at the decision point of a Y maze [54]. Sirota et al., 2008 showed that the pathway was critical in cognitive pathways. Thus the finding that the theta rhythm in this pathway is specifically impaired in the animal model of 22q.11.2 copy number deletion, the most penetrating genetic form of schizophrenia [21], indicates that this pathway may be a pivotal point for drug action. The pathway is highly stress-sensitive [1,19,20,26], with LTP being abolished by stress. H-PFC theta, and consequently nested gamma, coherence is crucial for cognitive tasks and for maintaining context [55]; clear phase locking of H-PFC rhythms has been demonstrated [53,56,57]. Clozapine increases theta rhythm in frontal cortex in conscious rats [30,31]. and reverses the effects of PCP above 5 Hz in this model [32]. Furthermore, increases in theta in frontal cortex in man predict antipsychotic efficacy of clozapine better than plasma levels [33]. In conclusion, the unique electrophysiological effects of EGIS 11150 support its unique profile in cognitive tests [25].

Nevertheless, some reservations must be made about H-PFC connectivity. Coherence values are susceptible to effects of reference electrode and volume conduction of sources, resulting in non-independence of signal recorded at separate electrode locations. [37,58,59]. However, the changes in the H-PFC connection occur at lower concentrations of EGIS 11150 compared to H-sensorimotor cortex, thus volume transmission cannot account for these changes, which are also concordant with the effects on H-PFC LTP. Furthermore, a battery of different findings is compatible with effects of EGIS 11150 on coherence in this pathway.

EGIS 11150 also increased theta rhythm in the CA1 hippocampal region in slice preparations. This is a true theta rhythm as it was blocked by gabazine, as well as by an I_h_ blocker. Gamma rhythm was increased, gamma rhythm could not be measured in the in vivo experiments, but it is normally embedded in the theta rhythm. The pharmacology of modulation of theta rhythm in slice preparations has been described [60]. EGIS 11150 induced theta rhythm in the CA1 region, with CA3 being resistant, in conditions where prazosin (or the agonist phenylephrine) was inactive: activity in slice preparations indicates direct pharmacological effects.

EGIS 11150 was more potent than currently used antipsychotic agents in reversing the effects of PCP. Aghajanian and Marek [61,62], showed in vitro that PCP, acting as a propsychotic agent and blocking the normal throughput of glutamate-NMDA receptors, releases glutamate onto AMPA receptors in the prefrontal cortex, and these effects can be modulated by 5-HT_2A_-receptors and by a_1_-adrenoceptors. This explains some, but not all, of the effectiveness of EGIS 11150 in this paradigm. Phencyclidine- and ketamine-induced psychosis in animals or man are relevant models for schizophrenia and no compound (other than EGIS 11150) is known to fully reverse PCP [39]. Furthermore, EGIS 11150 restored social interactions in rats treated with PCP, whereas olanzapine did not [25,46]. PCP-induced prepulse inhibition and behavioural effects were blocked at the same doses, indicating a real potential for therapeutic efficacy [25]. However, EGIS 11150 was less effective against dopaminergic stimuli, consistent with its lowered D_2_ affinity and low propensity to cause catalepsy [25,46]. Inhibitors of glycine transporter-1 have been shown to have similar effects changes in PCP-induced spectral power in rat prefrontal cortex [63] attributed to changes in extracellular dopamine and glycine levels [64].

The α_1_-adenoceptor potency is important as Lim et al. [65] have shown that locus coeruleus stimulation modulated H-PFC LTP. Phenylephrine, by activating α_1_-adrenoceptors in layer V pyramidal neurones of cerebral cortex, suppresses AMPA-mediated excitatory postsynaptic currents [66], and AMPA-mediated transmission is necessary in the presence of NMDA-induced dysfunction. Noradrenaline induces long-term depression (LTD) in rat prefrontal cortical neurones and, with stress, downregulates PFC function via a_1_-adrenoreceptors, with an involvement of NMDA receptors [66]. Thus α_1_-adrenoreceptor antagonism is necessary, but may confer some measure of postural hypotension in healthy volunteers in phase I clinical studies.

Consistent with the effects on theta rhythm and the restoration of LTP after stress, EGIS 11150 showed robust procognitive effects in the behavioural tests at the same low doses as the electrophysiological activity was observed, indicating an antipsychotic agent with a marked procognitive potential [25]. In the present study we extended these findings and showed that EGIS 11150 was effective at restoring reversal learning impaired by a course of treatment with phencyclidine. It is well established that the PFC is essential for reversal learning [67,68]. The ability of EGIS 11150 to fully restore H-PFC LTP after stress at the low dose of 0.01 mg/kg i.p. is remarkable, as the only other drug reported to fully restore LTP is the atypical antidepressant tianeptine [19], or clozapine, in vitro [42] or in a mouse model of 22q11.2 deletion [69]. Stress has a very robust negative effect on neuronal plasticity in H-PFC, by impairing neurotrophic mechanisms, via glucocorticoid release [70]. In the present study we demonstrated that previous exposure to a platform stress even impaired LTP in ex vivo hippocampal slices prepared from animals exposed to platform stress, and that EGIS 11150 fully reversed this inhibition, even when added to the slice preparations. Thus the effects of EGIS 11150 are direct and not mediated via reflex adaptations.

EGIS 11150 (10µM) had no effect on allosteric modulation of NMDA receptors in binding studies, where ketamine fully displaced ^3^H-MK-801 binding and thus these interactions must be indirect [71]. Nevertheless, the effects of EGIS 11150 were mainly on glutamatergic rather than cholinergic theta, being atropine resistant, with marked effects on H-PFC coherence. The exact mechanism(s) are unknown, but the compound was found in a deliberate screening process for this profile, with good blood-brain-barrier penetration and pharmacokinetics, and with reduced effects on QTc, compared to risperidone. Novel screens can therefore find compounds with very novel pharmacology. Although it may appear that testing ~20 compounds, previously optimised for good pharmacokinetic properties with varied receptor profiles, in in vivo electrophysiology may seem labour-intensive it may be useful way to find genuinely novel compounds.

EGIS 11150 has excellent pharmacokinetics in rats and man, with ~70% bioavailability and a half-life of ten hours in man, representing the first agent found by screening to remedy dysconnectivity in specific brain regions relevant in schizophrenia, albeit a certain measure of postural hypotension was found in volunteers in phase I.

## Figures and Tables

**Table 1:** Details of preparations, animals, and doses used.

Preparation, Location, Lead Author	Animals, Weight Age, *n*	Ethical Approval	Drug, Dose, Concentration
2.3 Hippocampal slice preparation: oscillations, in vitro Newcastle, UK; Cunningham M. O.	Adult male Wistar rats (~150 g) and aged male (8 months) *n* = 6 (EGIS; CA1) *n* = 6 (EGIS; CA3) *n* = 5 (ZD7288) *n* = 5 (gabazine) *n* = 3 (atropine)	UK Animals Act 1986 & European Union Directive2010/63/EU (No. 25308)	Isoflurane (6–8 mls), Xylazine (~10 mg/kg i.m.), Ketamine (~100 mg/kg i.m.) EGIS 11150 (200 nM), ZD7288 (10 µM), gabazine (2 µM), atropine (100 nM)
2.4 EEG Electrode implantation, in vivo Paris, France; Sebban C.	Conscious rats Adult male Wistar rats (~150 g) and aged male (8 months) *n* = 6/group, 1 animal/cage One or Two-way ANOVA (see text)	The institutional guidelines (Centre de recherche scientifique) and the prerogatives from the French Agriculture and Forestry Ministry (decree 874848, licence A91429)	Chloral hydrate (350 mg/kg i.p.) EGIS 11150 (0.02–0.01–0.05 mg/kg) Clozapine 0.3 mg/kg (s.c.) Risperidone 0.3 mg/kg (i.p.) Olanzapine 5 mg/kg (i.p.) PCP 1 mg/kg (i.p.) Drugs dissolved in 0.9% NaCl
2.5 Inhibition of H-PFC LTP caused by platform stress, in vivo Paris, France; Jay T. M.	Adult male Srague Dawley rats (300–400 g) *n* = 6/non stressed NaCl, stressed EGIS11150 and stressed Hal *n* = 8/stressed NaCl and stressed clozapine	All animal experiments were performed in accordance with our institution guidelines (Centre National de la Recherche Scientifique) and the prerogatives from the French Agriculture and Forestry Ministry (decree 874848, license A91429).	Pentobarbital (60 mg/kg i.p.) EGIS11150 (0.01 mg/kg, i.p.), clozapine (0.3 mg/kg, I.p.) or Haloperidol (1 mg/kg, i.p.)
2.6 Mouse hippocampal slice preparation, Influence of stress on LTP, in vitro Dundee, Scotland; Lambert J. J.	2–4 month-old male C57BL/6 mice (22–25 g) *n* = 4–12	Schedule 1 of the UK Government Animals Act 1986 & Home Office project license PPL 60/3575	EGIS 11150 (100–300 nM)
2.7 Reversal learning in the rewarded T-maze, in vivo EGIS, Budapest, Hungary; Antoni F. A.	Male C57Bl/6j Mice (20–25 g) 5 animals/cage 40 animals 10/treatment group, all included in the results	Animal Care and Use Ethical Committee of Egis Pharmaceuticals PLC and complied with the Hungarian Law of Animal Care and Use (1998. XVIII) and Directive 2010/63/EU on the protection of animals used for scientific purposes.	PCP (3 mg/kg i.p.) or saline (i.p.) derived from previous studies with Egis 11150 (0.01 or 0.03 mg/kg p.o.) (Gacsályi, I. et al., 2013)

## Data Availability

The data are available from EGIS Pharmaceuticals Ltd., 1106 Budapest, Hungary and Institut de Recherches Internationales Servier, 92284 Suresnes, France, or from the authors listed in Table 1.

## Supplementary Materials

The following supporting information can be downloaded at: https://www.mdpi.com/article/10.3390/cells11071181/s1: Figure S1: No apparent effect of EGIS 11150 on reversal learning in intact mice. Detailed methodology of EEG analysis.

## References

[1] Agid Y., Buzsaki G., Diamond D.M., Frackowiak R., Giedd J., Girault J.-A., Grace A., Lambert J.J., Manji H., Mayberg H. (2007). How can drug discovery for psychiatric disorders be improved?. Nat. Rev. Drug Discov..

[2] Cohen J.D., Insel T.R. (2008). Cognitive Neuroscience and Schizophrenia: Translational Research in Need of a Translator. Biol. Psychiatry.

[3] Carter C.S., Barch D.M., Buchanan R.W., Bullmore E., Krystal J.H., Cohen J., Geyer M., Green M., Nuechterlein K.H., Robbins T. (2008). Identifying Cognitive Mechanisms Targeted for Treatment Development in Schizophrenia: An Overview of the First Meeting of the Cognitive Neuroscience Treatment Research to Improve Cognition in Schizophrenia Initiative. Biol. Psychiatry.

[4] Kerns J.G., Nuechterlein K.H., Braver T., Barch D.M. (2008). Executive Functioning Component Mechanisms and Schizophrenia. Biol. Psychiatry.

[5] Ochsner K.N. (2008). The Social-Emotional Processing Stream: Five Core Constructs and Their Translational Potential for Schizophrenia and Beyond. Biol. Psychiatry.

[6] Ranganath C., Minzenberg M.J., Ragland J.D. (2008). The Cognitive Neuroscience of Memory Function and Dysfunction in Schizophrenia. Biol. Psychiatry.

[7] Artigas F., Schenker E., Celada P., Spedding M., Lladó-Pelfort L., Jurado N., Núñez M., Santana N., Troyano-Rodriguez E., Riga M.S. (2016). Defining the brain circuits involved in psychiatric disorders: IMI-NEWMEDS. Nat. Rev. Drug Discov..

[8] Friston K., Frith C.D. (1995). Schizophrenia: A disconnection syndrome?. Clin. Neurosci..

[9] Stephan K.E., Friston K.J., Frith C.D. (2009). Dysconnection in schizophrenia: From abnormal synaptic plasticity to failures of self-monitoring. Schizophr. Bull..

[10] Pocklington A.J., Rees E., Walters J., Han J., Kavanagh D.H., Chambert K.D., Holmans P., Moran J., McCarroll S.A., Kirov G. (2015). Novel Findings from CNVs Implicate Inhibitory and Excitatory Signaling Complexes in Schizophrenia. Neuron.

[11] Young K.A., Manaye K.F., Liang C.-L., Hicks P.B., German D.C. (2000). Reduced number of mediodorsal and anterior thalamic neurons in schizophrenia. Biol. Psychiatry.

[12] Godsil B., Bontempi B., Mailliet F., Delagrange P., Spedding M., Jay T.M. (2015). Acute tianeptine treatment selectively modulates neuronal activation in the central nucleus of the amygdala and attenuates fear extinction. Mol. Psychiatry.

[13] Spedding M., Chattarji S., Spedding C., Jay T.M. (2021). Brain circuits at risk in psychiatric diseases and pharmacological pathways. Therapies.

[14] Park A.J., Harris A.Z., Martyniuk K.M., Chang C.-Y., Abbas A.I., Lowes D.C., Kellendonk C., Gogos J.A., Gordon J.A. (2021). Reset of hippocampal–prefrontal circuitry facilitates learning. Nature.

[15] Takashim A., Petersson K.M., Rutters F., Tendolkar I., Jensen O., Zwarts M.J., McNaughton B.L., Fernández G. (2006). Declarative memory consolidation in humans: A prospective functional magnetic resonance imaging study. Proc. Natl. Acad. Sci. USA.

[16] Bähner F., Meyer-Lindenberg A. (2017). Hippocampal–prefrontal connectivity as a translational phenotype for schizophrenia. Eur. Neuropsychopharmacol..

[17] Tamminga C.A. (2006). The neurobiology of cognition in schizophrenia. J. Clin. Psychiatry.

[18] Kupferschmidt D.A., Gordon J.A. (2018). The dynamics of disordered dialogue: Prefrontal, hippocampal and thalamic miscommunication underlying working memory deficits in schizophrenia. Brain Neurosci. Adv..

[19] Rocher C., Spedding M., Munoz C., Jay T.M. (2004). Acute Stress-induced Changes in Hippocampal/Prefrontal Circuits in Rats: Effects of Antidepressants. Cereb. Cortex.

[20] Spedding M., Neau I., Harsing L. (2002). Brain plasticity and pathology in psychiatric disease: Sites of action for potential therapy. Curr. Opin. Pharmacol..

[21] Sigurdsson T., Stark K.L., Karayiorgou M., Gogos J.A., Gordon J.A. (2010). Impaired hippocampal–prefrontal synchrony in a genetic mouse model of schizophrenia. Nature.

[22] Karayiorgou M., Simon T.J., Gogos J.A. (2010). 22q11.2 microdeletions: Linking DNA structural variation to brain dysfunction and schizophrenia. Nat. Rev. Neurosci..

[23] Spedding M. (2011). Resolution of controversies in drug/receptor interactions by protein structure. Limitations and pharmacological solutions. Neuropharmacology.

[24] Urban J., Clarke W., Von Zastrow M., Nichols D.E., Kobilka B., Weinstein H., Javitch J., Roth B.L., Christopoulos A., Sexton P. (2006). Functional Selectivity and Classical Concepts of Quantitative Pharmacology. J. Pharmacol. Exp. Ther..

[25] Gacsályi I., Nagy K., Pallagi K., Lévay G., Hársing L.G., Móricz K., Kertész S., Varga P., Haller J., Gigler G. (2012). Egis-11150: A candidate antipsychotic compound with procognitive efficacy in rodents. Neuropharmacology.

[26] Spedding M., Jay T., Silva J.C., Perret L. (2005). A pathophysiological paradigm for the therapy of psychiatric disease. Nat. Rev. Drug Discov..

[27] Buzsáki G. (2005). Theta rhythm of navigation: Link between path integration and landmark navigation, episodic and semantic memory. Hippocampus.

[28] Yamaguchi Y., Sato N., Wagatsuma H., Wu Z., Molter C., Aota Y. (2007). A unified view of theta-phase coding in the entorhinal–hippocampal system. Curr. Opin. Neurobiol..

[29] Seager M.A., Johnson L.D., Chabot E.S., Asaka Y., Berry S.D. (2002). Oscillatory brain states and learning: Impact of hippocampal theta-contingent training. Proc. Natl. Acad. Sci. USA.

[30] Sebban C., Zhang X.Q., Tesolin-Decros B., Millan M.J., Spedding M. (1999). Changes in EEG spectral power in the prefrontal cortex of conscious rats elicited by drugs interacting with dopaminergic and noradrenergic transmission. J. Cereb. Blood Flow Metab..

[31] Sebban C., Tesolin-Decros B., Millan M.J., Spedding M. (1999). Contrasting EEG profiles elicited by antipsychotic agents in the prefrontal cortex of the conscious rat: Antagonism of the effects of clozapine by modafinil. J. Cereb. Blood Flow Metab..

[32] Sebban C., Tesolin-Decros B., Ciprian-Ollivier J., Perret L., Spedding M. (2002). Effects of phencyclidine (PCP) and MK 801 on the EEGq in the prefrontal cortex of conscious rats; antagonism by clozapine, and antagonists of AMPA-, α1- and 5-HT2A -receptors. J. Cereb. Blood Flow Metab..

[33] Gross A., Joutsiniemi S.-L., Rimon R., Appelberg B. (2004). Clozapine-Induced QEEG Changes Correlate with Clinical Response in Schizophrenic Patients: A Prospective, Longitudinal Study. Pharmacopsychiatry.

[34] Tislerova B., Brunovsky M., Horáček J., Novak T., Kopecek M., Mohr P., Krajca V. (2008). LORETA Functional Imaging in Antipsychotic-Naive and Olanzapine-, Clozapine- and Risperidone-Treated Patients with Schizophrenia. Neuropsychobiology.

[35] Lisman J., Buzsaki G. (2008). A Neural Coding Scheme Formed by the Combined Function of Gamma and Theta Oscillations. Schizophr. Bull..

[36] Fujisawa S., Amarasingham A., Harrison M.T., Buzsáki G. (2008). Behavior-dependent short-term assembly dynamics in the medial prefrontal cortex. Nat. Neurosci..

[37] Sirota A., Montgomery S., Fujisawa S., Isomura Y., Zugaro M., Buzsáki G. (2008). Entrainment of Neocortical Neurons and Gamma Oscillations by the Hippocampal Theta Rhythm. Neuron.

[38] Krystal J.H., D’Souza D.C., Mathalon D., Perry E., Belger A., Hoffman R. (2003). NMDA receptor antagonist effects, cortical glutamatergic function, and schizophrenia: Toward a paradigm shift in medication development. Psychopharmacology.

[39] Lahti A.C., Weiler M.A., Tamara M., Parwani A., Tamminga C.A. (2001). Effects of Ketamine in Normal and Schizophrenic Volunteers. Neuropsychopharmacology.

[40] Jentsch J.D., Roth R.H. (1999). The neuropsychopharmacology of phencyclidine: From NMDA receptor hypofunction to the dopamine hypothesis of schizophrenia. Neuropsychopharmacology.

[41] Büttner-Ennever J. (1997). The Rat Brain in Stereotaxic Coordinates, 3rd edn. J. Anat..

[42] Zhang H., Etherington L.-A., Hafner A.-S., Belelli D., Coussen F., Delagrange P., Chaouloff F., Spedding M., Lambert J.J., Choquet D. (2012). Regulation of AMPA receptor surface trafficking and synaptic plasticity by a cognitive enhancer and antidepressant molecule. Mol. Psychiatry.

[43] Deacon R.M.J., Rawlins J.N.P. (2006). T-maze alternation in the rodent. Nat. Protoc..

[44] Gacsályi I., Móricz K., Gigler G., Megyeri K., Machado P., Antoni F.A. (2018). Persistent therapeutic effect of a novel α5-GABAA receptor antagonist in rodent preclinical models of vascular cognitive impairment. Eur. J. Pharmacol..

[45] Singer P., Boison D., Möhler H., Feldon J., Yee B.K. (2009). Deletion of glycine transporter 1 (GlyT1) in forebrain neurons facilitates reversal learning: Enhanced cognitive adaptability?. Behav. Neurosci..

[46] Gacsalyi I., Gigler G., Kompagne H., Haller J., Azar M.R., Moricz K., Levay G. (2007). Pharmacology of a new atypical antipsychotic agent, EGIS-11150/S36549. Schizophr. Bull..

[47] Gillies M.J., Traub R.D., LeBeau F.E.N., Davies C.H., Gloveli T., Buhl E.H., Whittington M.A. (2002). A Model of Atropine—Resistant Theta Oscillations in Rat Hippocampal Area CA1. J. Physiol..

[48] Chrobak J.J., Buzsáki G. (1998). Operational dynamics in the hippocampal-entorhinal axis. Neurosci. Biobehav. Rev..

[49] Jensen O., Tesche C.D. (2002). Frontal theta activity in humans increases with memory load in a working memory task. Eur. J. Neurosci..

[50] Sederberg P.B., Kahana M.J., Howard M.W., Donner E.J., Madsen J.R. (2003). Theta and Gamma Oscillations during Encoding Predict Subsequent Recall. J. Neurosci..

[51] Jay T.M., Witter M.P. (1991). Distribution of hippocampal CA1 and subicular efferents in the prefrontal cortex of the rat studied by means of anterograde transport of Phaseolus vulgaris-leucoagglutinin. J. Comp. Neurol..

[52] Tierney P.L., Dégenètais E., Thierry A., Glowinski J., Gioanni Y. Influence of the Hippocampus on Interneurons of the Rat Prefrontal Cortex. The European Journal of Neuroscience. https://pubmed.ncbi.nlm.nih.gov/15233760/.

[53] Colgin L.L. (2011). Oscillations and hippocampal–prefrontal synchrony. Curr. Opin. Neurobiol..

[54] Benchenane K., Peyrache A., Khamassi M., Tierney P.L., Gioanni Y., Battaglia F.P., Wiener S.I. (2010). Coherent Theta Oscillations and Reorganization of Spike Timing in the Hippocampal- Prefrontal Network upon Learning. Neuron.

[55] Jones M., Wilson M.A. (2005). Theta Rhythms Coordinate Hippocampal–Prefrontal Interactions in a Spatial Memory Task. PLoS Biol..

[56] Hyman J.M., Zilli E.A., Paley A.M., Hasselmo M.E. (2005). Medial prefrontal cortex cells show dynamic modulation with the hippocampal theta rhythm dependent on behavior. Hippocampus.

[57] Siapas A.G., Lubenov E.V., Wilson M.A. (2005). Prefrontal Phase Locking to Hippocampal Theta Oscillations. Neuron.

[58] Fein G., Raz J., Brown F.F., Merrin E.L. (1988). Common reference coherence data are confounded by power and phase effects. Electroencephalogr. Clin. Neurophysiol..

[59] Nunez P.L., Srinivasan R., Westdorp A.F., Wijesinghe R.S., Tucker D.M., Silberstein R.B., Cadusch P.J. (1997). EEG coherency: I: Statistics, reference electrode, volume conduction, Laplacians, cortical imaging, and interpretation at multiple scales. Electroencephalogr. Clin. Neurophysiol..

[60] Roopun A.K., Kramer M.A., Carracedo L.M., Kaiser M., Davies C.H., Traub R.D., Kopell N.J., Whittington M.A. (2008). Temporal interactions between cortical rhythms. Front. Behav. Neurosci..

[61] Aghajanian G.K., Marek G.J. (2000). Serotonin model of schizophrenia: Emerging role of glutamate mechanisms. Brain Res. Brain Res. Rev..

[62] Aghajanian G.K., Marek G.J. (1999). Serotonin, via 5-HT2A receptors, increases EPSCs in layer V pyramidal cells of prefrontal cortex by an asynchronous mode of glutamate release. Brain Res..

[63] Hársing L.G., Gacsalyi I., Szabo G., Schmidt E., Sziray N., Sebban C., Tesolin-Decros B., Matyus P., Egyed A., Spedding M. (2003). The glycine transporter-1 inhibitors NFPS and Org 24461: A pharmacological study. Pharmacol. Biochem. Behav..

[64] Nagy K., Marko B., Zsilla G., Matyus P., Pallagi K., Szabo G., Juranyi Z., Barkoczy J., Levay G., Harsing L.G. (2010). Alterations in Brain Extracellular Dopamine and Glycine Levels Following Combined Administration of the Glycine Transporter Type-1 Inhibitor Org-24461 and Risperidone. Neurochem. Res..

[65] Lim E.P., Tan C.H., Jay T.M., Dawe G.S. (2010). Locus coeruleus stimulation and noradrenergic modulation of hippocampo-prefrontal cortex long-term potentiation. Int. J. Neuropsychopharmacol..

[66] Kobayashi M., Sasabe T., Shiohama Y., Koshikawa N. (2008). Activation of α1-adrenoceptors increases firing frequency through protein kinase C in pyramidal neurons of rat visual cortex. Neurosci. Lett..

[67] McAlonan K., Brown V.J. (2003). Orbital prefrontal cortex mediates reversal learning and not attentional set shifting in the rat. Behav. Brain Res..

[68] Bartolo R., Averbeck B.B. (2020). Prefrontal Cortex Predicts State Switches during Reversal Learning. Neuron.

[69] Tripathi A., Spedding M., Schenker E., Didriksen M., Cressant A., Jay T.M. (2020). Cognition- and circuit-based dysfunction in a mouse model of 22q11.2 microdeletion syndrome: Effects of stress. Transl. Psychiatry.

[70] Qi H., Mailliet F., Spedding M., Rocher C., Zhang X., Delagrange P., McEwen B., Jay T.M., Svenningsson P. (2009). Antidepressants reverse the attenuation of the neurotrophic MEK/MAPK cascade in frontal cortex by elevated platform stress; reversal of effects on LTP is associated with GluA1 phosphorylation. Neuropharmacology.

[71] Huang L., Abuhamdah S., Howes M.-J.R., Dixon C.L., Elliot M.S.J., Ballard C., Holmes C., Burns A., Perry E.K., Francis P.T. (2008). Pharmacological profile of essential oils derived from Lavandula angustifolia and Melissa officinalis with anti-agitation properties: Focus on ligand-gated channels. J. Pharm. Pharmacol..

