# Supplementary material for: Phenotypical Screening on Neuronal Plasticity in Hippocampal-Prefrontal Cortex Connectivity Reveals an Antipsychotic with a Novel Profile
Source: Cells. 2022 Mar 31;11(7):1181. doi: 10.3390/cells11071181 (PMC8997950; doi:10.3390/cells11071181)
Supplement: Supplementary file 1 [file cells-11-01181-s001.zip › cells-1638917-supplementary.pdf]

SUPPLEMENTARY MATERIAL

**EEG analysis**

EEG signals for each electrode were amplified, filtered (anti-aliasing filters: 90 db/oct) and digitalized (63 points/s) for the fast Fourier Transform (FFT) which allowed calculation of the power of EEG components from 1 to 30 Hz with 1 Hz steps ($\mu V^2$). Six absolute power spectra of EEG signals (corresponding to each electrode) were computed over 2 s intervals for 60 minutes after the i.p. injection of vehicle. Power spectra were computed (1-30 Hz) and averaged for each minute of recording. Hertz by Hertz drug-induced power changes were evaluated by the ratio of power after injection of the drug/power after injection of vehicle.

Coherence was calculated between the hippocampal and the prefrontal cortex power spectral amplitudes. Coherence is the squared analogue of a correlation coefficient and evaluates in the Fourier transform the existence of a linear relation ship between two signals (x and y):

$$|\rho xy(f)|2 = \frac{|Sxy(f)2|2}{Sxx(f) \bullet Syy(f)}$$

where $|\rho xy(f)|2$ .. = coherence between x and y fro one frequency, and

*S*xx(f) and *S*yy(f) are spectral densities of x and y

In our methodology, this calculation allows to split the total power of the PFC into two spectra. One is coherent with the hippocampal activity and the second, remaining represents PFC activity which is independent of the hippocampal activity.

Supplementary Figure S1:

**SUPPLEMENTARY Figure :**
**No apparent effect of Egis-1150 on reversal learning in intact mice**

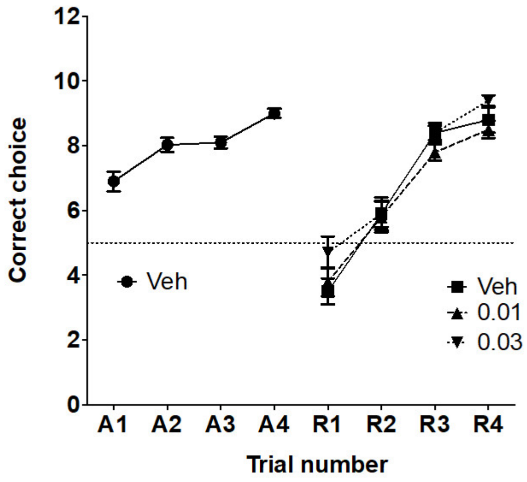


Lack of an effect of Egis 11150 (0.03 and 0.1 mg/kg p.o.) on reversal learning (right panel) in the rewarded T-maze by drug naïve mice. Data are mean ± SEM n=10/group.
